# Raisin Production by Convective–Microwave Dryer and Optimization of Ochratoxin A Content, Physicochemical, Sensory Characteristics, and Fungal Load of Raisin

**DOI:** 10.1002/fsn3.4661

**Published:** 2024-12-26

**Authors:** Majid Behfar, Ali Heshmati, Aghil Sharifzadeh, Maryam Taghdir, Sepideh Abbaszadeh

**Affiliations:** ^1^ Student Research Committee Baqiyatallah University of Medical Sciences Tehran Iran; ^2^ Department of Nutrition and Food Hygiene, Nutrition Health Research Center Hamadan University of Medical Sciences Hamadan Iran; ^3^ Department of Microbiology and Immunology, Faculty of Veterinary Medicine University of Tehran Tehran Iran; ^4^ Health Research Center, Life Style Institute Baqiyatallah University of Medical Sciences Tehran Iran; ^5^ Department of Nutrition and Food Hygiene, Faculty of Health Baqiyatallah University of Medical Sciences Tehran Iran

**Keywords:** drying processing, microwave power, ochratoxin A, optimization, raisin

## Abstract

Raisins are so popular in the human diet as a nutritional and sweet snack. The quality of this foodstuff depends on drying conditions. To minimize ochratoxin A (OTA) content and yeast and mold content (YMC) in raisins with favorable physicochemical and sensory properties, the response surface methodology (RSM) and the face‐centered central composite design (FCCD) were utilized. The independent variables were studied over a range of microwave power (0–220 W) and temperature air (35°C–55°C). The microwave power had a significant effect on the OTA value and YMC of dried samples. The use of microwaves in drying contributed to a higher decline in OTA content and YMC than in conventionally dried samples. The regression models detected for OTA content, acidity, pH, rehydration ratio (RR), water holding capacity (WHC), shrinkage, appearance, texture, acceptability, and YMC were significant and reliable. The desirability function was applied to recognize an optimized situation. The result of the RSM analysis showed the optimum conditions for obtaining healthy raisins with desired physicochemical and sensory attributes by microwave‐convective dryer at 220 W microwave power and 38.68°C air temperature.

## Graphical abstract

To minimize OTA content and YMC in raisin with favorable physicochemical and sensory properties, the response surface methodology (RSM) and the face‐centered central composite design (FCCD) were utilized. The result of RSM analysis showed the optimum conditions for obtaining healthy raisins with desired physicochemical and sensory attributes by microwave‐convective dryer at 220 W microwave power and 38.68°C air temperature.

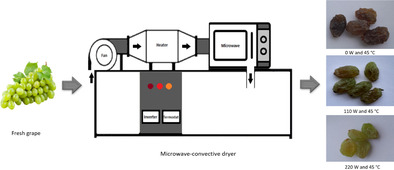

## 1 Introduction

Grapes due to their high economic value and health benefits are one of the most momentous garden crops (Ashtiani et al. 2020; International Organisation of Vine and Wine 2021). However, microbiological spoilage caused by high moisture and sugar content in grape berries is an undeniable problem (Ashtiani et al. 2020). Fortunately, raisin production reduces the moisture content to below 20%, which is a key technology for preventing fresh grape losses (Ashtiani et al. 2020; Cui et al. 2021). Raisin, as healthy and tasty, is one of the most favorite dried fruits worldwide (Cui et al. 2021). World production of dried grape was estimated at 1.4 million metric tons in 2021. Iran is the world's fourth‐largest raisin manufacturer after Turkey, the United States, and China produce 131,000 tons (International Organisation of Vine and Wine 2021). The anticancer, anti‐inflammation, and anti‐cardiovascular disease activity of dried vine fruit phenols have been proven in previous studies (Eftekhari, Alizadeh, and Ebrahimi 2012). Also, raisin is a nutritious snack containing carbohydrates, minerals, and antioxidant compounds (Cui et al. 2021).

Despite all the mentioned benefits of dried grape, the presence of ochratoxin A (OTA) in raisin has fascinated the researcher's attention as a global concern because this toxic fungal secondary metabolite menaces the health of raisin consumers (Hajok et al. 2019; Heshmati, Vahidinia, and Jafari 2013). Although around 400 basic mycotoxins have been recognized, OTA is one of the most potent carcinogens (Alhamoud et al. 2019), so the International Agency for Research on Cancer has considered this mycotoxin in the 2B carcinogens group (Organization and Cancer 1993). Also, OTA is a common and stable toxin, which is widely present in food, feed and human blood (Hajok et al. 2019; Wang et al. 2022). OTA is accepted as a cumulative mycotoxin and human frequent exposure to it through the diet leads to various complications such as hepatotoxins, nephrotoxins, neurotoxins, and immunotoxins. (Alhamoud et al. 2019; Wei et al. 2021).

Approximately 20 species of the genera Aspergillus and Penicillium were known as OTA generators (Maman et al. 2021). However, Aspergillus carbonarius is the principal source of OTA contamination in grape and related products. Because the majority of its strains produce large amounts the mycotoxin (Bragulat et al. 2019), Aw value of 0.95–0.99 and temperatures at 22°C–32°C is suitable conditions for secretion of OTA by Aspergillus carbonarius (Maman et al. 2021). Nowadays, the European Union for reducing the health hazards related to the ingestion of OTA and ensuring food safety in international trade considered regulatory limits (Bragulat et al. 2019; Wei et al. 2021). The maximum residue levels (MRLs) are similar across different dried grape forms. OTA limit by authorities in dried vine fruit (currant, raisin, and sultana) has been set at 8 μg/kg (The European Commission 2022, August 5).

The customary method of producing raisin is not effective for totally destroying OTA in the final product. In this regard, the occurrence of this mycotoxin in dried grape in several countries has been reported. For example, Heshmati, Vahidinia, and Jafari (2013) investigated the OTA level in the raisin processed by Hamadan workhouses; the average concentration of toxin was 1.72 μg/kg. But, OTA content in 5 of 66 samples (7.58%) was above the national standard level (5 μg/kg) (Heshmati, Vahidinia, and Jafari 2013). In another study, 50 dried grape samples marketed in various stores in Istanbul traditional bazaar were analyzed for OTA content; contamination abundance was 8% with mean concentrations of 1.15 μg/kg (Akdeniz, Ozden, and Alpertunga 2013).

There is a possibility of contamination of this product with yeast and mold content (YMC) within the steps of harvesting, maintenance, drying, forwarding, and sales (Karami et al. 2023). There are enough spore cells after drying and they can remain viable for months and cause various diseases (Alp and Bulantekin 2021). Hence, the presence of YMC in dried grape is concerning. A recent paper shows that the raisin supplied at Kabul city food markets is contaminated to YMC in 240 CFU/g and is need to be controlled (Jafari 2022). It seems that the lack of balance between the relative humidity and moisture content created a condition suitable moisture environment for mold growth (Alp and Bulantekin 2021). Therefore, eliminating YMC on grape during post‐harvest processing is vital to assure microbial safety and prolong the shelf life of raisin. Microwave ray treatment affects microbial enzyme activity and metabolism, and it is thought in combination with a convective dryer has the potential power for YMC decline (Deng et al. 2021).

Foodstuff contamination to OTA has caused heavy economic losses to manufacturers and exporters (Wang et al. 2022). On the contrary, the usual thermal processing used to inactivate microorganisms not be adequate for complete OTA degradation (Alkadi and Altal 2019). More temperature too causes noticeable deterioration of the valuable nutrients of dried grape (Cui et al. 2021). Hence, the variety of techniques for OTA removal in products food have become a focus of investigators (Wang et al. 2022). These strategies can be classified as physical, chemical, and biological. By far, the physical procedure has been the most impressive idea for mycotoxin dissipation from contaminated foodstuff (Jalili, Jinap, and Noranizan 2010).

The microwave heating is a physical method of preserving foodstuffs that have widely been applied in the food drying processes with the benefits of lower processing time, quality preservation, and energy efficiency (Alkadi and Altal 2019; da Silva et al. 2016). Although, Wanasirakul et al. (2014) observed an 84.56% reduction in the OTA value of white raisin after heated in microwave at 400 W for 60 s (Wanasirakul et al. 2014). However, Studies on the drying effects on YMC, OTA level, sensory, and physicochemical properties of dried grape should be studied together to be able to set up comprehensive protocols and good manufacturing practice (GMP) for raisin.

Sun drying, shade drying, and mechanical drying are used for drying commercial raisins (Wang et al. 2021). In sun drying, grapes are traditionally spread on the ground and exposed to the sun and natural air for 14–21 days, but shade drying protects the grapes from direct sunlight and the drying process lasts for nearly 15 days (Qin et al. 2020). These customary methods are slow and dependent on weather conditions that lead to microbial and insect contamination in raisin (Khiari, Zemni, and Mihoubi 2019). Mechanical drying technologies include fluidized bed dryers, freeze, vacuum, radiation, and conventional dryers (tray, tunnel, and drum) (Alp and Bulantekin 2021). Progressed drying methods for grape drying are most used in the food industry and interest to enhance the drying rate and improve raisin quality (Wang et al. 2021). On the contrary, combined dryers have gained interest because of their potential for safety and final product quality (Deng et al. 2021).

Thus, this study aimed to: (a) investigate the impact of microwave‐convective dryer conditions, that is, microwave power and temperature air on OTA content, YMC, physicochemical and sensory characteristics of raisin; (b) determine the optimum conditions for manufacturing healthy raisin with desirable physicochemical and sensory attributes; (c) creation the valid prediction models for each response; and (d) comparison of results between hot air convection dryer and microwave‐convective dryer.

## 2 Materials and Methodology

### 2.1 Materials

Thompson seedless grape ( *Vitis vinifera* ) samples were been purchased at a fruit shop in Hamedan. OTA standard with purity > 99% and phosphate buffer (PB) was provided by Sigma‐Aldrich (St. Louis, MO, USA). Toluene, sodium hydroxide, phosphate buffer saline (PBS), methanol, acetonitrile, acetic acid, and potato dextrose agar with analytical grade were obtained from Merck Inc. (Darmstadt, Germany). The ultrapure water was prepared with A Millipore Milli‐Q purification system (Millipore, Milford, CT, USA).

### 2.2 Grape Drying Process

**FIGURE 1:**
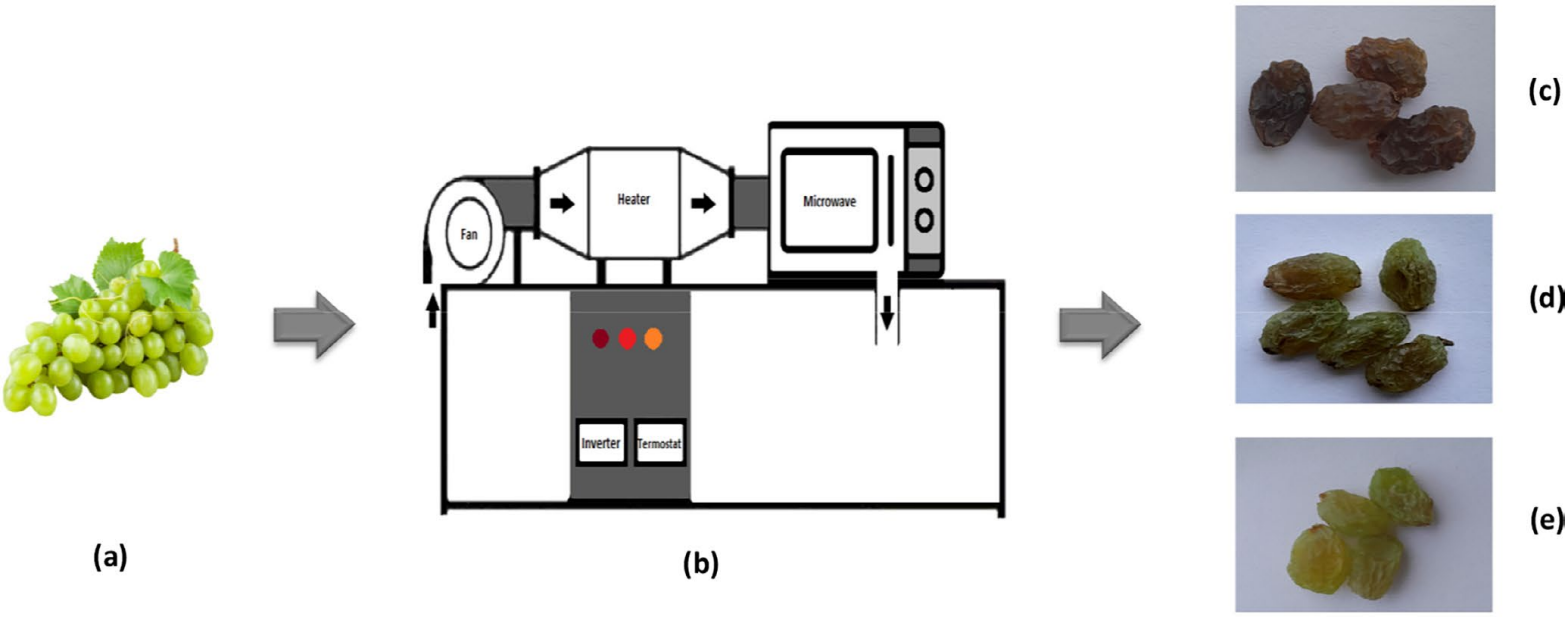
Raisin production process with microwave‐convective dryer: (a) Fresh grapes. (b) Microwave‐convective dryer analysis. (c) Raisin dried at 0 W and 45°C. (d) Raisin dried at 110 W and 45°C. (e) Raisin dried at 220 W and 45°C.

The OTA concentration was measured in high‐quality samples after manually sorting grapes. The mycotoxin level of fresh grapes was lower than our method limit of quantification (LOQ). In this regard, to help identify and understand, the degradation of OTA after drying from grape contaminated to the artificial toxin at a concentration of 8 μg/kg was used. This amount was chosen on the basis of the maximum residue limit for dried grapes by EFSA (The European Commission 2022, August 5). Drying trials were done using a modified hot‐air microwave dryer so that its temperature air and microwave power were controllable (Figure 1b). A thermostat equipped with a type k thermocouple was used to adjust the inlet air temperature at 35°C, 45°C, and 55°C levels. Three microwave power levels at 0, 110, and 220 W were manufactured by a microwave device (Panasonic NN‐ST757W, China) with 1100 W maximum power. Sample weight had taken by a digital balance (AND GF‐6000, Japan) in every 60 s. About 0.4 kg grape contaminated to 8.36 ± 1.52 μg/kg of OTA in each run was placed into a dryer chamber. When the moisture of the grape attained, the equilibrium step (without change in three sequential weightings) drying was ended.

### 2.3 OTA Extraction

Briefly, 4 g of ground raisin or mashed grape was added to 20 mL of methanol. Before being passed via filter paper (Whatman No. 1), the solution was mixed with a magnetic stirrer (HS15‐03P, Korea) for 2 min to create a homogeneous slurries suspension. Then, the aliquot 6 mL was diluted with 34 mL of PBS and passed via a conditioned immunoaffinity column (Puri‐Fast OTA IAC, Libios, France). After the column was eluted with 10 mL PBS at a 1 mL/min flow rate, they were dried in the air within 2 min. Then, 0.5 mL methanol under gravity was applied to leach OTA from IAC. Eventually, an extract of 100 μL was injected to the HPLC (Mozaffary, Milani, and Heshmati 2019).

### 2.4 OTA Measurement Validation

The linearity, accuracy, precision, and sensitivity were assessed in order to validate the method of OTA analysis. The signal‐to‐noise ratios of 3:1 as limit of detection (LOD) and 10:1 as LOQ were considered to identify the procedure sensitivity. Recovery in the three spike levels of 2.5, 5, and 10 μg/kg was examined to determine the accuracy of measurement. The recovery measurement was repeated on the same day (intra‐day precision) and three successive days (inter‐day precision). A calibration curve at 0.1–6.5 μg/kg levels was constructed using the peak area ratio of OTA versus its concentration.

### 2.5 HPLC Characteristics

The HPLC apparatus (Waters E2695; Waters Corporation, USA) was armed with a fluorescence detector and a reversed phase C18 column (ODC) (200 mm × 4.6 mm, i.d., 3 μm). The mobile phase composed of methanol, acetonitrile, water, and acetic acid (30:39:30:1, v/v/v/v) had a 1 mL/min flow rate. The wavelengths of excitation and emission of detector for OTA were 333 and 477 nm, respectively.

### 2.6 Shrinkage Degree Determination

The toluene transposition method was utilized to measure shrinkage. Five dried grapes were immersed in some toluene. The displaced toluene mass was determined with a laboratory scale and then divided by its density to obtain the sample volume. The shrinkage percentage was calculated through the following formula (Ashtiani et al. 2020):

$$\text{Shrinkage}\;\left(\%\right)=\frac{\left(\text{volume of grape}-\text{volume of raisin}\right)}{\text{volume of grape}}\times 100$$

### 2.7 Measurement Rehydration Ratio

Dried grape (2 g) was immersed in 100 mL distilled water. The weighing was performed every 5 min up to attaining the mass transfer balance. Following balance, the samples came out of the water and weighed afresh. The rehydration ratio (RR) was calculated as a down equation (Piskov et al. 2020):

$$\mathrm{RR}\;\left(‐\right)=\frac{\left(\text{weight of rehydrated raisin}-\text{weight of dried grape}\right)}{\text{weight of dried grape}}\times 100$$

### 2.8 Determination of Water Holding Capacity

About 1 g raisin was vortexed in 30 mL of distilled water for 2 min. The resulting dispersions were kept at room temperature for 30 min and centrifuged at 2682 ***g*** for 20 min. The tube with sediment was reweighed after draining off the supernatant. Water holding capacity (WHC) was obtained by the equation (Sridhar and Charles 2020):

$$\mathrm{WHC}\;\left(g/g\right)=\frac{\left(\text{sediment fresh weight}\;\left(g\right)-\text{sediment}\;\mathrm{dry}\;\text{weight}\;\left(g\right)\right)}{\text{sediment}\;\mathrm{dry}\;\text{weight}\;\left(g\right)}\times 100$$

### 2.9 Total Acidity and pH Analysis

Initially, 2 g of dried grape in 18 mL distilled water with a rotor‐stator homogenizer was homogenized. The suspension was used in order to pH measurement using a pH meter (Denver Instruments, UB—10) (Tontul and Topuz 2017). The water 20 mL was added to 10 mL of suspension and titrated with sodium hydroxide of 0.1 N to pH 8.1. Total acidity was gained according to the below formula (Mazlum and Nizamlioğlu 2021):

$$\text{Total acidity}\;\left(\%\right)=\frac{\text{the consumed NaOH amount}\times 0.007505}{\text{sample weight}\;\left(g\right)}\times 100$$

### 2.10 YMC Analysis

Briefly, 25 g raisin with 225 mL of PB in the sterile bottle was blended for 2 min using a rotor stator homogenizer. The specimens were diluted in PB serially and distributed on potato dextrose agar. The plates at 25°C for 5 days were incubated, and the YMC was enumerated (CFU/g) (McCoy et al. 2015).

### 2.11 Sensory Attributes Investigate

Twelve experienced panelists (six men and six women aged between 25 and 50 years) of students and university‐trained staff were selected. The panelists got preparatory guidance related to descriptive sensory properties profiles such as taste, appearance, texture, and acceptability before the 10‐point hedonic method. All dried vine fruit samples were presented and evaluated at once a day. Tap water was fed to persons between samples to neutralize the previous specimen effect and cleanse the mouth. Finally, the panelists were demanded to devote a numerical value between 1 and 10 where 1 indicates hate intense and 10 depicts fond intense (Wang et al. 2017).

### 2.12 Modeling and Data Statistical Analysis

Response surface methodology (RSM) is an applied statistical tool to describe the relation between variables and identify the process's optimum condition. The face‐centered central composite design (FCCD) with two factors on three levels was utilization. This design consisted of 13 experimental treatments with five replicates at the central point. The replicate at the center of the design was used to determine the accuracy. Various parameters could affect the response, but power and temperature were considered as the most influential. The microwave power (0–220 W) and air temperature (35°C–55°C) were the factors used. The upper and lower limits of independent variables were selected on the basis of the past literature and preliminary trials. The result's statistical analysis was conducted via the software Design‐Expert version 7.0.0. The results were statistically tested at the levels of probability *p* < 0.01 and *p* < 0.05. Analysis of variance to investigate the competence of the regression equation was performed by indicators such as the *f* value, *p* value, and *R* ^2^. The experimental data were matched to the second‐order polynomial equation comes as:

(1) $$Y=\beta_{0}+\beta_{1}X_{1}+\beta_{2}X_{2}+\beta_{11}X_{1}^{2}+\beta_{22}X_{2}^{2}+\beta_{12}X_{12}$$

where *Y* implies the response, *β*_0_ is a constant term, *β*_1_ and *β*_2_ are the linear terms, *β*_11_ and *β*_22_ are the quadratic terms, and *β*_12_ is the interaction terms. *X*_1_ and *X*_2_ represent microwave power and temperature air, respectively.

### 2.13 Optimization by Desirability Function

The optimal level of independent variables for one response might be far from the optimal condition for other responses. The desirability function‐based multi‐objective optimization was performed to identify the best value of variables to obtain a desirable solution in compliance with the selected criteria. The desirability function represents how desirable the responses are at selected levels of parameters, which range from 0 to 1. The main criterion of research present is healthy raisin generation, which is required to OTA value and YMC be minimized. The second criterion is acceptance of the product by the consumer, which must be to RR, WHC, and sensory characteristics score be maximized. In addition, shrinkage has to a minimum. The independent variables, pH, and acidity were considered within range.

## 3 Results and Discussion

### 3.1 Method Validation Data

Method validation parameters are given in Table 1. The relative standard deviation (RSD) for repeatability assay was ranged from 3.54% to 16.62%. The recovery values at the three defined levels varied from 87.82% to 103.51%. According to the Commission Regulation (EC) No. 401/2006, the recovery values should be within 70%–110% and RSD ≤ 20 (Commission 2006). The LOQ and LOD were 1.02 and 0.33 μg/kg, respectively. In addition, the linearity of the calibration curve was 0.9985. From the results of this study and previous literature can be concluded that HPLC is a sensitive, rapid, and accurate way for OTA evaluation (Sakin et al. 2018).

**TABLE 1:** Parameters of method validation to quantify OTA in grape and raisin.

Product	Spiking level (μg/kg)	Recovery		Linearity			Sensitivity	
		Intra‐day ± RSD (%)	Inter‐da ± RSD (%)	Range (μg/kg)	Equation	*R* ^2^	LOD (μg/kg)	LOQ (μg/kg)
Grape	2.5	87.82 ± 6.96	95.3 ± 11.61	0.1–6.5	*Y* = 0.9016*X* + 2.1691	0.9985	0.33	1.02
	5	95.93 ± 15.45	91.41 ± 16.62					
	10	103.51 ± 6.94	98.08 ± 3.54					
Raisin	2.5	101.31 ± 4.28	94.3 ± 10.29					
	5	99.97 ± 7.88	89.16 ± 5.79					
	10	88.39 ± 12.34	94.95 ± 5.61					

### 3.2 The Impact of Microwave Dryer on OTA

The standard deviation (SD) for OTA content was 0.21. This low value indicates a good fitting and high precision of the model. In addition, the *R* ^2^ value of 0.9696 emphasizes the correlation between the experimental and predicted data (Table 3). The second‐order polynomial equation was used to draw a relationship between independent variables and OTA amount as:

(2) $$Y_{\mathrm{OTA}\;\text{value}}=4.30-1.10X_{1}+0.76X_{1}^{2}$$

As depicted in Table 3, the microwave energy has more impact on OTA level compared with temperature due to its higher *f* value (168.22) and lower *p* value (*p* < 0.01), so the lowest OTA value was observed after most power (3.89 μg/kg). Alkadi and Altal (2019) explained the great efficacy of high‐level power of microwave oven in the decontamination of OTA due to the food geometrical dimension, which influences decreasing the penetration depth of the ray (Alkadi and Altal 2019). The grape spiked with 8.36 ± 1.52 μg/kg mycotoxin was used for raisin generation. The OTA amount after drying was reduced to lower than 8 μg/kg (the maximum residue limit determined by European Union legislation). Destruction of the C–C double bond of the lactone ring of the dihydro‐isocoumarin moiety is a mechanism for detoxification of OTA by microwave because the OTA toxicity is due to lactone ring binding properties with DNA and protein (Zhang et al. 2021).

### 3.3 The Impact of Microwave Dryer on Physicochemical Properties

#### 3.3.1 The Impact of Microwave Dryer on Total Acidity and pH

The quadratic model for total acidity and liner model for pH were statistically significant (*p* < 0.05; 0.01), as noted in Table 3. In addition, their lack of fit was insignificant (*p* > 0.05). Due to the models that were insignificant compared with pure error, these regression equations can be effectively used to estimate the total acidity and pH of the specimens on the basis of factor changes. Total acidity and pH presented the regression equation as:

(3) $$Y_{\text{Total acidity}}=1.33-0.085X_{1}+0.083X_{2}$$

(4) $$Y_{\mathrm{pH}}=4.04+0.062X_{1}-0.097X_{2}$$

**FIGURE 2:**
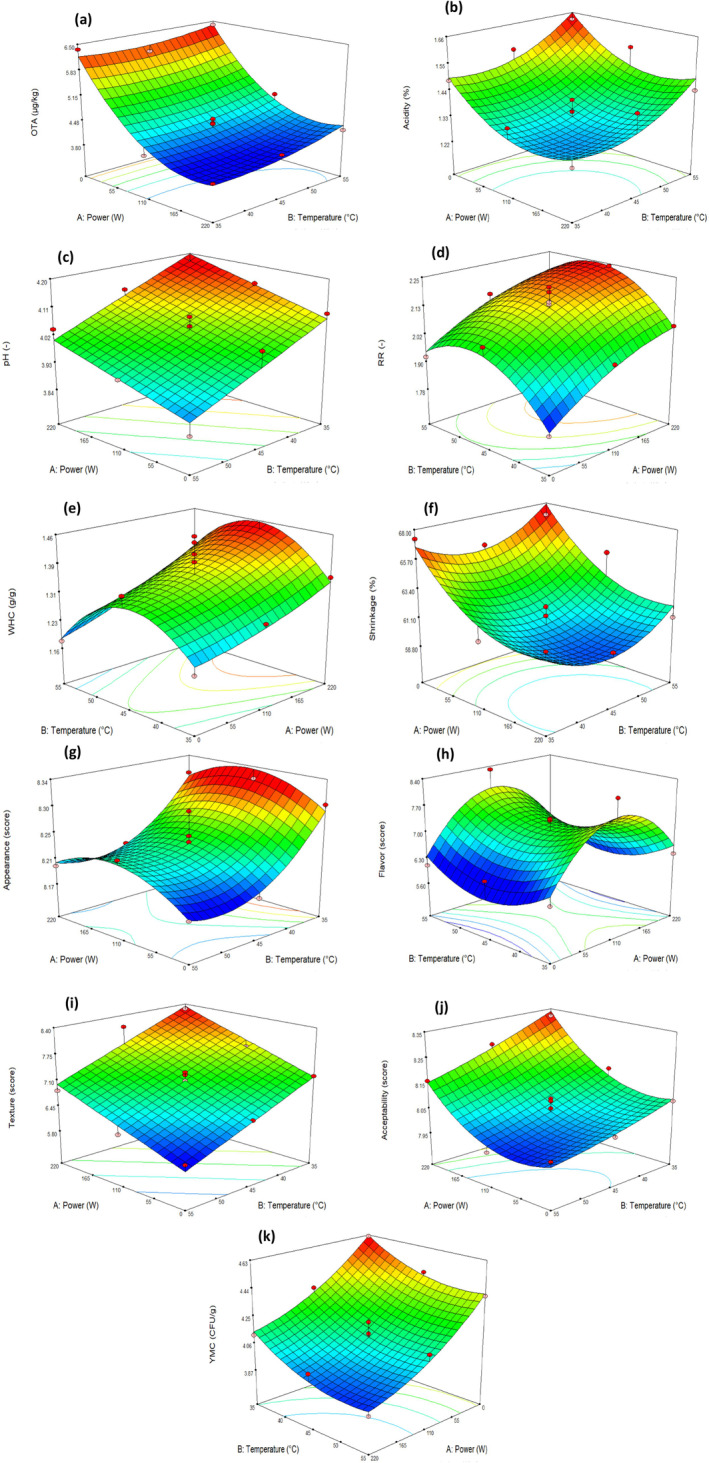
Effect of temperature and power on OTA (a), physicochemical properties (b–f), sensory characteristics (g–j), and YMC (k) at 3D plots.

Grape acidity is correlated with the presence of tartaric, malic, and ascorbic acids (Kapłan et al. 2021; Lokhande, Ranveer, and Sahoo 2017). The total acidity of dried vine fruit at treatment of 55°C and without microwave was 1.63%, whereas in maximum radiation, it was 1.44% presented in Figure 2b. Perhaps reason for this decreased molecular vibration is caused by microwave energy, which leads to tearing the grape skin and oxidation of ascorbic acid with oxygen (Carranza‐Concha et al. 2012). However, Baysal et al. (2015) have reported contradictory results. Malic acid of microwave‐dried apple slices at 360 W after 20 min was significantly higher from the no ray samples (Baysal et al. 2015).

#### 3.3.2 The Impact of Microwave Dryer on RR

According to Table 3, The *f* value of 19.77 for a model fitted to RR displays that the equation is significant. The linear effects of independent variables and the temperature quadratic effects significantly influenced RR (*p* < 0.01), whereas the rest terms were nonsignificant (*p* > 0.05). After removing the insignificant effects, the estimated regression coefficients are reported below:

(5) $$Y_{\mathrm{RR}}=2.16+0.12X_{1}+0.067X_{2}-0.13X_{2}^{2}$$

RR is an index that expresses how much dehydrated fruit can come back to its original by rehydration process, and the higher RR content is considered as the quality desired of dried fruit (Berk and Taşkın 2023; İzli and Yildiz 2021). When drying grape at 35°C–45°C, RR rose due to drying in less time, and less irreversible physicochemical changes occurred. However, an opposite trend was observed when drying at 45°C–55°C, as this maybe a result of the intense shrinkage and texture collapse produced by higher air temperatures leading to a decrement in RR. The RR enhanced as the power level increased owing to shorter drying time (Figure 2d). The intercellular gaps created by microwave energy allow water to return into pores and give rise to an increased RR (Askari, Emam‐Djomeh, and Mousavi 2006). Lokhande, Ranveer, and Sahoo (2017) illustrated that as the drying power rose from 350 to 1000 W, the RR of raisin was 1.40%–2.50% enhancement (Lokhande, Ranveer, and Sahoo 2017).

#### 3.3.3 The Impact of Microwave Dryer on WHC

Variance analysis in Table 3 indicates the microwave power compared with air temperature because the *f* values higher than 1 have more precision. Furthermore, the *p* value less than 0.05 implies that this variable is significant. The equation for WHC as a function of coded factors can be represented in the following form:

(6) $$Y_{\mathrm{WHC}}=1.37+0.068X_{1}-0.11X_{2}^{2}$$

WHC of raisin during the process varied from 1.18 g/g at 0 W and 55°C to 1.44 g/g at 110 W and 45°C as given in Table 2. WHC that affects the functional properties of the foodstuff was raised during the drying process with enhancing microwave power (Figure 2e). This trend is due to the high inner pressure manufactured by microwave heating, which can result in the structure of dried vine fruit expanding and puffing (Ghanem et al. 2012). The data regarding the impact of microwave energy on WHC in past the literature are different. For instance, Jafari, Goli, and Toghyani (2022) indicated that with raised the microwave power from 200 to 700 W, the WHC of egg powder was incremented as 3.07 to 4.17 mL/g (Jafari, Goli, and Toghyani 2022). However, some authors observed that the WHC of dried apples linearly declined when the level of microwave incident power increased up to 10 W/g (Bilbao‐Sáinz, Andrés, and Fito 2005).

**TABLE 2:** Experimental values of responses and factor levels used in FCCD design.

Run	Factors				Responses										
	Power (W)		Temperature (°C)		OTA (μg/kg)	Physicochemical properties					Sensory characteristics				YMC (CFU/g)
	Actual	Coded	Actual	Coded		Acidity (%)	pH (−)	RR (−)	WHC (g/g)	Shrinkage (%)	Appearance (score)	Flavor (score)	Texture (score)	Acceptability (score)	
1	220	+1	35	−1	3.89	1.29	4.17	2.05	1.35	61.99	8.31	6.42	8.27	8.32	4.12
2	220	+1	45	0	4.06	1.42	4.12	2.24	1.42	60.09	8.21	6.48	8.10	8.25	3.99
3	110	0	45	0	4.41	1.35	3.99	2.21	1.39	60.11	8.23	7.37	7.01	8.05	4.13
4	110	0	35	−1	4.04	1.36	4.14	1.97	1.28	60.95	8.32	8.29	7.59	8.15	4.34
5	0	−1	45	0	5.98	1.55	4.03	2.04	1.35	65.62	8.18	6.19	6.56	8.01	4.45
6	110	0	45	0	4.23	1.31	4.01	2.14	1.44	61.29	8.24	6.41	7.31	8.08	3.97
7	110	0	45	0	4.52	1.22	4.02	2.08	1.35	62.02	8.29	7.35	6.89	8.02	4.21
8	0	−1	35	−1	6.37	1.48	4.09	1.78	1.21	67.29	8.30	6.12	7.21	8.08	4.62
9	110	0	45	0	4.38	1.40	4.08	2.15	1.29	59.98	8.19	6.35	7.09	7.95	4.05
10	110	0	55	+1	4.77	1.56	3.94	2.12	1.24	65.00	8.24	8.31	6.21	7.95	4.12
11	0	−1	55	+1	6.40	1.63	3.84	1.92	1.18	67.01	8.18	6.10	6.02	8.00	4.39
12	110	0	45	0	4.05	1.24	4.05	2.19	1.41	58.87	8.25	7.31	7.24	8.09	4.10
13	220	+1	55	+1	4.21	1.44	4.04	2.16	1.38	61.17	8.20	6.39	6.84	8.16	3.87

#### 3.3.4 The Impact of Microwave Dryer on Shrinkage

The adj‐*R* ^2^ of 0.7551 suggested that the total variation of 75% for shrinkage was attributed to the parameters. Meanwhile, low CV (2.25%) reveals the good precision and reliability of the actual values (Table 3). To predict the independent variables effect on the shrinkage, the second‐order polynomial equation was generated:

(7) $$Y_{\text{shrinkage}}=60.59-2.78X_{1}+2.03X_{2}^{2}$$

**TABLE 3:** ANOVA evaluation of coded factors for responses.

Source of variance	OTA	Physicochemical properties					Sensory characteristics				YMC
		Acidity	pH	RR	WHC	Shrinkage	Appearance	Flavor	Texture	Acceptability	
Total model	44.72^a^	4.29^b^	31.51^a^	19.77^a^	5.25^b^	8.40^a^	5.80^b^	2.76^ns^	52.62^a^	12.02^a^	17.22^a^
*X*_1_	168.22^a^	6.44^b^	18.23^a^	44.18^a^	11.23^b^	23.56^a^	0.73^ns^	0.36^ns^	44.44^a^	31.13^a^	62.19^a^
*X*_2_	4.52^ns^	6.19^b^	44.79^a^	14.02^a^	0.11^ns^	0.74^ns^	19.36^a^	4.187E‐004^ns^	60.80^a^	14.71^a^	13.91^a^
*X*_12_	0.49^ns^	0.00^ns^	—	0.12^ns^	0.36^ns^	0.037^ns^	0.030^ns^	6.979E‐005^ns^	—	0.73^ns^	0.017^ns^
*X*_1_^2^	36.62^a^	3.59^ns^	—	2.30^ns^	0.23^ns^	5.14^ns^	5.07^ns^	13.20^a^	—	10.44^b^	2.71^ns^
*X*_2_^2^	1.26^ns^	1.93^ns^	—	26.40^a^	13.56^a^	5.81^b^	7.07^b^	3.33^ns^	—	0.15^ns^	3.47^ns^
Lack‐of‐fit	1.69^ns^	1.46^ns^	1.00^ns^	0.42^ns^	0.39^ns^	1.72^ns^	0.15^ns^	1.66^ns^	1.86^ns^	0.28^ns^	0.37^ns^
*R* ^2^	0.9696	0.7538	0.8631	0.9339	0.7895	0.8572	0.8055	0.6632	0.9132	0.8957	0.9248
Adj‐*R* ^2^	0.9480	0.5780	0.8357	0.8866	0.6392	0.7551	0.6665	0.4226	0.8959	0.8211	0.8711
Pred‐*R* ^2^	0.8104	−0.0839	0.7460	0.7982	0.2810	0.1137	0.5550	−0.4922	0.8489	0.6895	0.7959
C.V. (%)	4.40	5.85	0.88	0.044	3.76	2.25	0.35	8.73	2.95	0.58	1.83
Std. dev.	0.21	0.082	0.035	2.10	0.050	1.40	0.029	0.60	0.21	0.047	0.077
Adq‐Precision	18.143	6.193	18.632	15.044	7.927	8.625	7.922	5.206	24.584	11.454	13.961

*Note:* X_1_ = Power; X_2_ = Temperature.

^a^ *p* < 0.01.

^b^ *p* < 0.05.

^ns^ Not significant.

In the drying step, the replacement of the air with intercellular water causes a pressure imbalance in tissue and tensions in the structure of the cells, resulting in shrinkage (Kaveh et al. 2021). Shrinkage of food products is considered an unfavorable change in the quality characteristics of the dehydrated fruit. The customer has a negative mentality relative to the differences in shape, volume reduction, and hardness increment of wrinkles products (İzli and Yildiz 2021). As shown in Figure 2f, microwave power has negative and significant effects on the shrinkage (*p* < 0.01). This finding is in agreement with the research of Kaveh et al. (2021). They had seen the highest shrinkage of pomegranate (70.21%) at 270 W microwave energy and the lowest amount (50.5%) at 630 W microwave energy (Kaveh et al. 2021). In contrast, it has been reported that the shrinkage of nectarine slices increased from 51.09% to 62.27% due to rise of microwave power from 80 to 320 W (Miraei Ashtiani, Sturm, and Nasirahmadi 2018). The drying in various temperatures nonsignificantly affected the shrinkage. Some authors observed no significant differences in the shrinkage of the quince samples between temperatures 60°C, 70°C, and 80°C (*p* > 0.05) (İzli and Yildiz 2021).

### 3.4 The Impact of Microwave Dryer on Sensory Properties

When consumers select a foodstuff, the sensory properties have the most influence on them. Hence, the raisin quality was reflected in the four major characteristics of appearance, flavor, texture, and overall acceptability. ANOVA evaluation's results of sensory attributes are given in Table 3. The first‐order and second‐order coefficients of temperature on appearance were significant (*p* < 0.05; 0.01). Therefore, the quadratic regression model for the appearance of samples is adequate as follows:

(8) $$Y_{\text{Appearance}}=8.24-0.052X_{2}+0.046X_{2}^{2}$$

The grapes dried by microwave and hot air were greener than those dried by hot air only (Figure 1c–e). Similarly, it was observed that the dried white cherry by microwave‐hot air due to its lighter color and resemblance color with the non‐dried white cherry is satisfactory (Berk and Taşkın 2023). This shows that microwave‐hot air dryer makes the water elimination process more steady, gentle, and wide which precludes the external layer from hardening and dark color (Qu et al. 2019; Soysal et al. 2009). However, the panelists could not perceive the effect of microwave power to appearance of raisin, as a group of the assessors preferred darker raisin and the other group preferred lighter sample.

The insignificant regression equation and the low coefficient of determination (*R* ^2^ = 0.6632) for the flavor reveal that the mathematical model could not fit with the experimental results properly for this response (Table 3). The flavor in our study is the only response that RSM was not successful in developing a predictive model for that.

To check the model adequacy fitted to the texture, the *R* ^2^ was used. The predicted *R* ^2^ is 0.8489 and is in reasonable agreement with their corresponding adjusted *R* ^2^ as the difference is < 0.2 (Table 3). Thus, this is an indicator the model is significant. The first‐order model for the optimization of texture is a function of power and temperature can be exposed in the following form:

(9) $$Y_{\text{Texture}}=7.10+0.57X_{1}-0.67X_{2}$$

Figure 2i shows texture score of treatments declined significantly with enhancing air temperature from 35°C to 55°C (*p* < 0.01). In justification of this trend, Langová et al. (2020) explained that drying the surface of the grape due to high temperature leads to poor migration of water from inside and the formation of a stiff surface layer (Langová et al. 2020). Table 2 illustrates that dehydration at 35°C and 220 W was the most suitable trial for texture acceptance as it took the highest score from panelists. In microwave‐assisted drying, microwave energy is absorbed mostly by water in the product and is spent to evaporate water. Therefore, heat is not transferred to the dried texture and remains intact (Sunjka et al. 2004).

The coefficient of variation (CV) for overall acceptance is 0.58. This small value expresses a better precision and reliability of the experiments performed. Additionally, the calculated *R* ^2^ is 0.8957, which can resulted in the model being capable of explaining 89.57% of the variability in the acceptability score. As such, the polynomial equation for that comes as:

(10) $$Y_{\text{Acceptability}}=8.04+0.11X_{1}-0.073X_{2}+0.091X_{1}^{2}$$

The acceptability score in run 1 due to the use of low temperature and high power was higher than in other runs (Table 2). Soysal et al. (2009) found that drying the red peppers at microwave low energy (597.20 W) received the highest score in overall acceptance that is contrasted with our findings (Soysal et al. 2009).

Drying by microwave ray depending on the type of way and the food material can have favorable and/or detrimental effects (Luka et al. 2024). Sunjka et al. (2004) stated that dried sample by microwave vacuum has a tough texture, caramelized odor, and burnt taste (Sunjka et al. 2004). Yet, the use of hot‐air companion microwave energy in the present research overcame the nonuniform heating and led to top‐quality foodstuff production.

### 3.5 The Impact of Microwave Dryer on YMC

According to the signal‐to‐noise ratio greater than four for YMC (Adeq Precision = 13.961%), which indicates the presence of an adequate signal, it can result in the below polynomial model is in accordance with experimental results:

(11) $$Y_{\mathrm{YMC}}=4.11-0.25X_{1}-0.12X_{2}$$

The using of microwave in dried grape samples decreased their YMC compared with that of the control samples so that the highest YMC (4.62 CFU/g) was observed in the control sample at 35°C and the lowest (3.87 CFU/g) YMC was found in the sample treated by 220 W microwave at 55°C (Table 2). The microwave irradiation reduced the YMC of raisin to below the allowable counts recommended by the Food and Drug Administration (FDA). In confirmation of our results, Nejad (2003) showed that drying in the microwave oven at 1000 W leads to the perfect elimination of YMC in saffron (Nejad 2003). The moisture removal caused by microwave appears to create porosity, which leads to more transmitted heat and YMC decline (Luka et al. 2024).

### 3.6 Optimization Analysis

**FIGURE 3:**
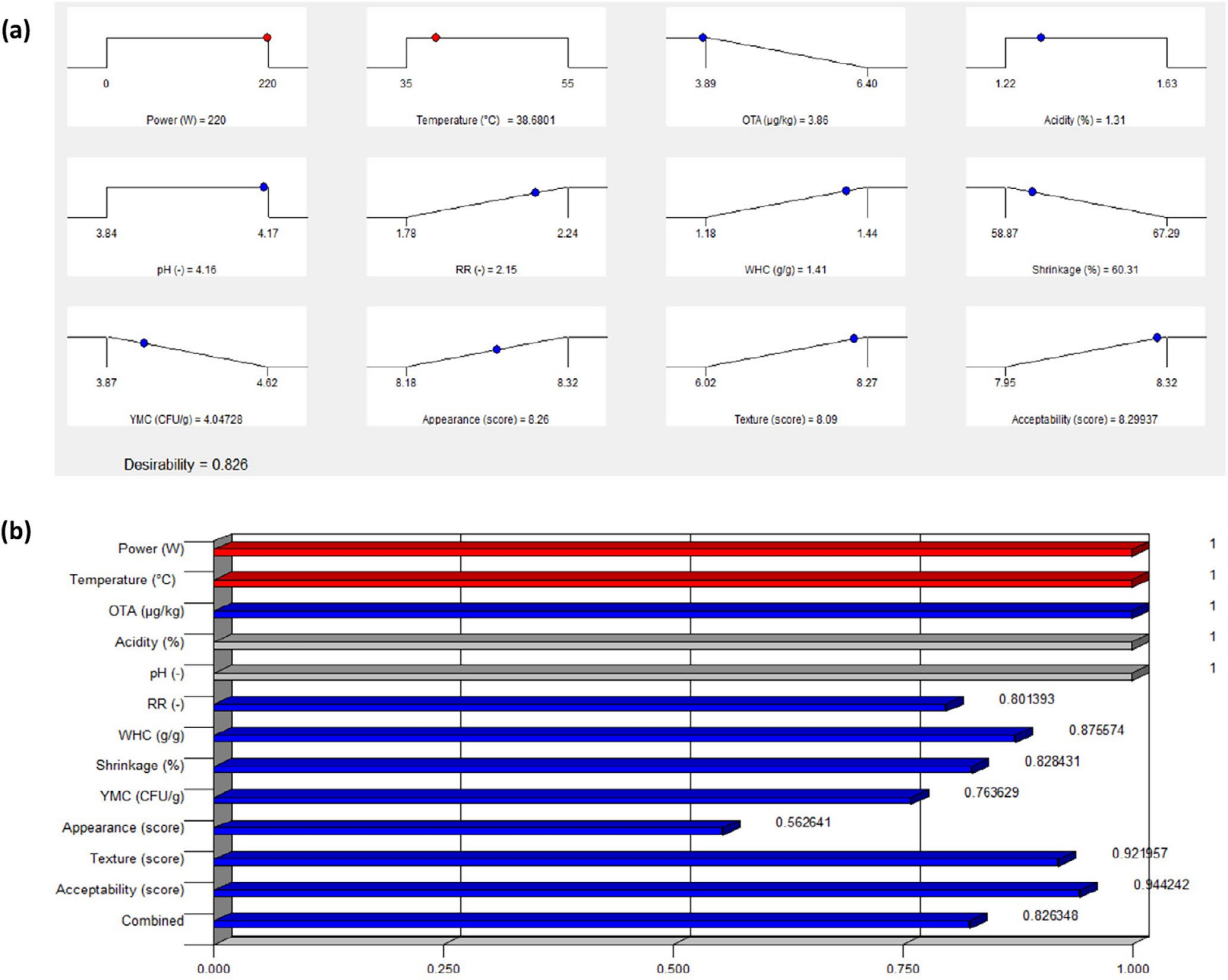
The optimum conditions of raisin production: (a) Predicted values at optimal point. (b) Desirability values of different variables.

In order to produce safe raisins with suitable sensory and physicochemical properties, multi‐objective optimization was used. The optimal levels for independent and dependent variables are displayed in Figure 3a. The 220 W power and 38.68°C air temperature are the experimental conditions of optimum predicted by FCCD. This point leads to the desired levels of the responses with desirability of 82.6%. Figure 3b depicts individual and combined desirability values of variables. The desirability function for parameters, acidity, and pH is equal to one because they were considered in range in the optimization, becomes more ideal response value as the desirability amount increases. In this regard, the desirability function for OTA with amount of one is the most satisfying response.

## 4 Conclusion

RSM was known as an efficient statistical instrument to model the impact of microwave power and air temperature on OTA level, YMC, physicochemical, and sensory characteristics of raisin. By using this method, the valid predictive equations and optimum condition of the dryer were identified. The high coefficient of determination accepts an adequate mathematical description of proposed models so that the changes in the OTA content, acidity, RR, WHC, shrinkage, appearance, acceptability, and YMC were adequately described by quadratic models. Moreover, the selected linear models had fitted well to the experimental data of pH and texture. Microwave‐assisted drying, compared with hot air drying alone, significantly reduced OTA content and YMC. Furthermore, sensory evaluation demonstrated the dried grape was acceptable by the consumer panel. The most important obstacle applicate microwave dryer equipment in the industry is the high investment expense. However, the combination of microwave dryer with hot air both reduces the process cost and improves drying efficiency.

## Author Contributions

**Majid Behfar:** investigation (equal), data curation (equal), methodology (equal), software (equal), formal analysis (equal), validation (equal), writing – original draft (equal). **Sepideh Abbaszadeh:** supervision (equal), conceptualization (equal), investigation (equal), project administration (equal), review and editing (equal). **Ali Heshmati:** supervision (equal), software (equal), funding acquisition (equal). **Maryam Taghdir:** supervision (equal), methodology (equal). **Aghil Sharifzadeh:** conceptualization (equal), methodology (equal), resources (equal).

## Conflicts of Interest

The authors declare no conflicts of interest.

## Data Availability

All demanded data during this study are in this published article.
